# Inhibition of the bioavailability of heavy metals in sewage sludge biochar by adding two stabilizers

**DOI:** 10.1371/journal.pone.0183617

**Published:** 2017-08-23

**Authors:** Zhujian Huang, Qin Lu, Jun Wang, Xian Chen, Xiaoyun Mao, Zhenli He

**Affiliations:** 1 College of Natural Resources and Environment, South China Agricultural University, Guangzhou, China; 2 Guangdong Provincial Key Laboratory of Environmental Pollution Control and Remediation Technology, Guangzhou, P. R. China; 3 Indian River Research and Education Center, Institute of Food and Agricultural Sciences, University of Florida, Fort Pierce, FL, United States of America; RMIT University, AUSTRALIA

## Abstract

Agricultural application of sewage sludge (SS) after carbonization is a plausible way for disposal. Despite its benefits of improving soil fertility and C sequestration, heavy metals contained in sewage sludge biochars (SSB) are still a concern. In this study, two types of heavy metal stabilizers were chosen: fulvic acid (FA) and phosphogypsum (with CaSO_4_, CS, as the main component). The two stabilizers were incorporated into SS prior to 350°C carbonization for 1 h at the rates of 1%, 2%, or 4%. The obtained SSBs were then analyzed by Fourier transform infrared spectroscopy (FTIR) and X-ray photoelectron spectroscopy (XPS). Total and available concentrations of four heavy metals, i.e., Zn, Pb, Cd, and Ni, in the SSBs were determined. In addition, a series of pot soil culture experiments was conducted to investigate the effects of stabilizers incorporation into SSB on heavy metal bioavailability and the uptake by plants (corn as an indicator) and plant biomass yield, with SS and SSB (no stabilizers) as controls. The results showed that incorporation of both FA and CS increased functional groups such as carboxyl, phenol, hydroxyl, amine and quinine groups in the SSBs. The percentage of heavy metals in sulfuric and oxidizable state and residual state of SSBs were significantly increased after carbonization, and hence the mobility of the heavy metals in SSBs was decreased. The introduction of the stabilizers (i.e., FA or CS) significantly lowered the total and available concentrations of Zn, Pb, Cd, and Ni. The reduction in available heavy metal concentration increased with incorporation rate of the stabilizers from 1% to 4%. In the treatments with FA or CS incorporated SSB, less heavy metals were taken up by plants and more plant biomass yields were obtained. The mitigating effects were more pronounced at higher rates of FA or CS stabilizer. These findings provide a way to lower bioavailability of heavy metals in SS or SSB for land application or horticulture as a peat substitute.

## Introduction

With increasing population and urbanization, huge amounts of sewage sludge (SS) are produced every day. In China, approximately 12.53 million tons of SS is produced each year [1]. Disposal and/or beneficial utilization of the ever increasing SS have become a challenge worldwide. Recently, SS carbonization has been considered as an environmentally friendly way of SS treatment. The resulting product, SS biochar (SSB), can be used as soil amendment to increase soil organic matter, improve soil fertility, and remediate polluted soils by heavy metals or organic contaminants [2,3]. Recently, it was found that SSB can been explored on as a peat substitute for growing media components, which can increase the N, P and K content of growing media [3]. However, SS itself contains heavy metals and therefore, SSB usually contains more heavy metals than biochars made from plant residues [4]. As metals are non-biodegradable; they may be released from SSB, taken up by plants, and amplify along the food chains, and eventually pose a threat to ecosystem functions and/or human health. Therefore, the heavy metals in SSB need to be stabilized to minimize their environmental risk.

Common metal stabilizers include zeolite [5], red mud, apatite [6], sepiolite [7], fly ash [8], iron/manganese oxides [1], phosphates, limestone and Ca-rich materials [9]. The mechanisms of stabilization include surface adsorption, precipitation, formation of stable complexes, and ligands or ion exchange [10]. For example, phosphate rock immobilizes Pb from aqueous solutions and soils through the formation of solid pyromorphite-like minerals [11].

Studies have shown positive effects of stabilizer application on lowering heavy metal availability in contaminated soils. But little research has been conducted to investigate the effects of stabilizers on heavy metal availability in SSB. In this study, two types of stabilizers (fulvic acid, FA, and calcium sulfate, CS), incorporated at different rates, were investigated for their effects on the available concentrations of four heavy metals (i.e., Zn, Pb, Cd, and Ni) in SSB. In addition, a soil pot experiment was conducted to investigate effect of SSB with stabilizer incorporation on heavy metal uptake by plants and plant biomass yield.

## Materials and methods

### Materials and apparatus

The sewage sludge (SS) is secondary sludge (excess activated sludge out of system) obtained aerobic treatment of sludge from the secondary sedimentation tank of the activated sludge system in Guangzhou Liede domestic sewage treatment plant (using improved A^2^/O process) in Guangzhou, Guangdong Province, China. We have got the permission from the managers of domestic sewage treatment plant. After transported to the laboratory, the SS was air-dried for 2 d, oven-dried at 60 ^o^C to constant weight, ground and passed through a 40-mesh sieve prior to use. The organic stabilizer used in this study was weathered coal. Since FA is the main component of the weathered coal [12], hereafter. FA is used to stand for this stabilizer. The inorganic stabilizer was phosphogypsum with CaSO_4_ (CS) as the main component. Hereafter, CS is used to stand for this stabilizer. Phosphogypsum (also named ardealite) is a by-product of the phosphate fertilizer industry. It is formed by the chemical attack of the phosphate rock withesulphuric acid to produce phosphoric acid. This waste is generally stored in piles near the fertilizer factory, whose main ingredient is gypsum (chemical component is CaSO_4_) [13]. The radioactive activity (176 Bq·kg^-1^) of the phosphogypsum we used is safe for using as building materials in China (GB 6566–2010). The quantity of the phosphogypsum added to the soil is very low, and also safe in the application of soil amendment. The soil used in the pot experiment was collected from the campus of South China Agricultural University. It is uduits, with a pH of 6.2, a CEC of 8.35 C mol·Kg^-1^, an EC of 156 uS/cm (s/w = 1:5), an organic matter content of 0.5%,and available N, P, and K of 33 mg N/kg, 6 mg P/kg, and 50 mg K/kg, respectively. The organic matter content and EC of SS were 53.02% and 1030 uS/cm (s/w = 1:5), respectively. The heavy metal concentrations of the soil, SS and FA are shown in **Table 1**.

**Table 1:** Heavy metal concentrations (mg/kg) of the soil, sewage sludge (SS), fulvic acid (FA) and phosphogypsum (CS).

	Zn		Pb		Cd		Ni	
	Total	Available	Total	Available	Total	Available	Total	Available
Soil	70.11	7.24	32.78	1.30	0.45	0.09	16.90	0.26
SS	764.20	354.54	119.70	28.71	3.00	1.42	49.90	22.65
FA	29.72	10.23	11.88	2.55	0.21	0.03	4.64	0.92
CS	31.21	13.72	29.07	3.36	0.28	0.06	1.39	0.05

Fourier transform infrared (FTIR) spectra were recorded between 4000 cm^-1^ and 400 cm^-1^ using a Hitachi EPI-G2 infrared spectrophotometer with DTGS KBr detector. The number of scans is 32, the resolution is 4 cm^-1^ and scan rate is 1.928 cm^-1^ step^-1^. The samples were mixed with KBr at the ratio of 1:180 and pelletized. The X-ray photoelectron spectra (XPS) were measured with an ANELVA AES-430S X-ray photoelectron spectrometer and the binding energy of C 1s was shifted to 284.6 eV as an internal reference.

### SSB preparation

The two stabilizers were added to the oven dried and sieved SS at the rates of 1%, 2%, and 4% on a dry weight basis. After thoroughly mixed, the mixtures were carbonized at 350 ^o^C for 1 h. The carbonization temperature of 350 ^o^C was selected because our previous study [1] showed that the stabilizers were most effective in reducing availabilities of the heavy metals at this temperature. The loss of thermal conversion for SS samples are shown in **S1 Fig** (Supporting Information), and at 350 ^o^C for 1 h, the weight loss is approximately 20%. The obtained biochars were labeled as SSBFA1, SSBFA2, and SSBFA4 for FA incorporation at 1%, 2%, and 4%, respectively and as SSBCS1, SSBCS2, and SSBCS4 for CS incorporation at 1%, 2%, and 4%, respectively.

### Heavy metal uptake by plants

**Fig 1:**
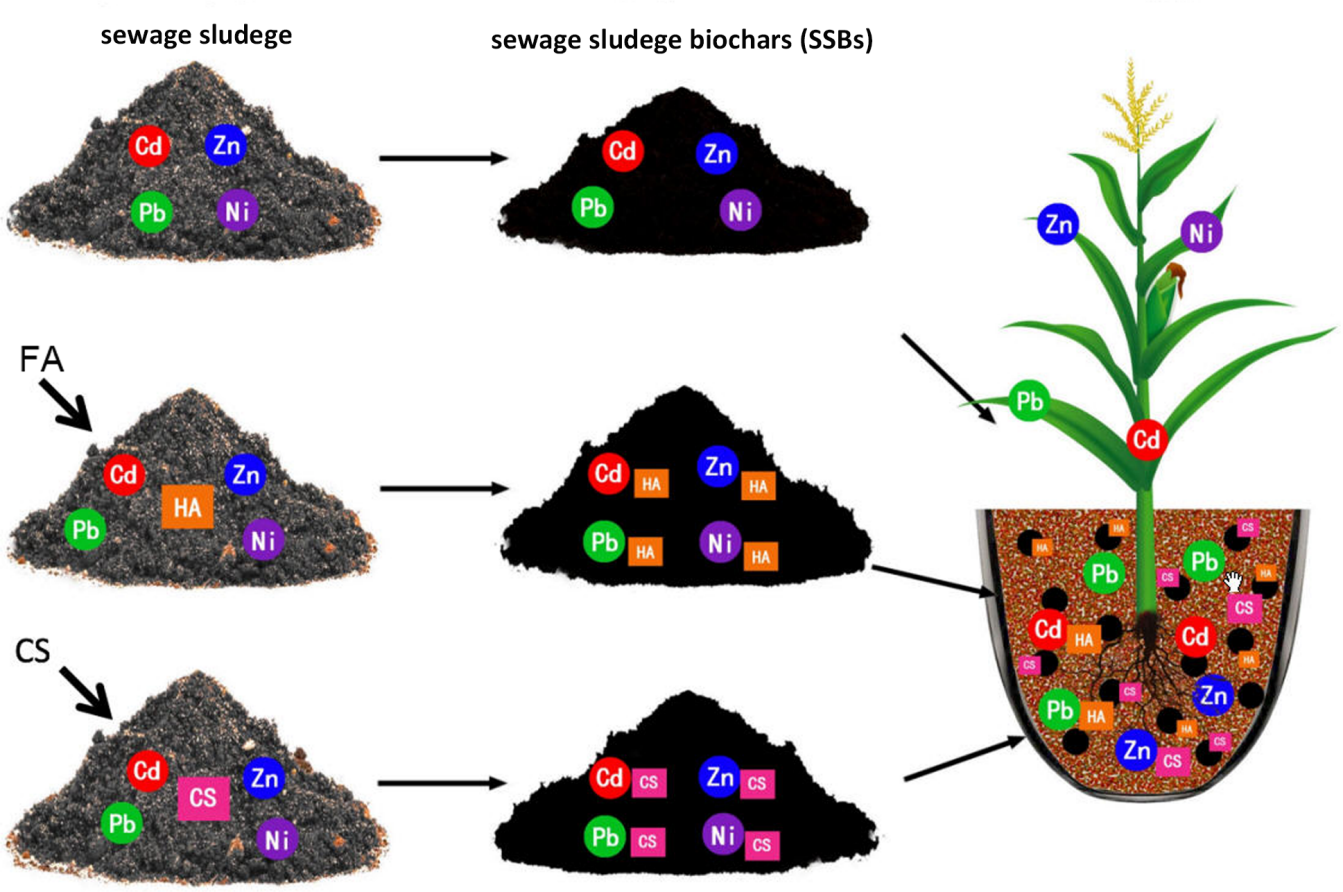
Schematic representation of the experiment design.

The soil culture experiment was conducted in the SCAU greenhouse in Guangzhou (113.368926E, 23.16368N) with the natural daylight (the light intensity 20–300 lx during the daytime). The temperatures during the pot study are between 25–36°C, and moisture was adjusted to 65% of field holding capacity. In pot experiments, a total of 7 treatments were set up in four replicates: CK, SS, SSB, SSBFA2, SSBFA4, SSBCS2, and SSBCS4. For CK, no SS or SSB was applied. For treatment SS, SS but not SSB was applied. For the other five treatments, the corresponding SSB was applied. For each pot, 4 kg air dried soil was used (The size of pot: the upper diameter is 20 cm, the lower diameter is 15 cm, the height is 18 cm, and 18 kg soil/pot); sewage sludge or biochars were applied at 0.5% on a dry weight basis; basic fertilizers of 1.13 g urea, 0.65 g ammonium dihydrogen phosphate and 0.8 g potassium chloride were applied. Soil, sewage sludge/biochar, and fertilizers were mixed thoroughly before being put into each pot, and moisture was adjusted to 65% of field holding capacity. The design of this study can be intuitively displayed in **Fig 1**.

On August 30^th^, 2013, 3 seeds of waxy corn (*Zea mays* L. ceratina Kulesh) were sown in each pot. At the 3^rd^ day after germination, the seedlings were thinned with only 2 seedlings left in each pot. During the whole experiment, the plants were watered once per day with 200 ml water/pot for the first 20 days and twice per day with 300 ml water each time at the later stage. The aboveground parts of the corn plants were harvested at the 45^th^ day after germination. Both fresh and oven dried weights of the harvested corn plants were recorded. To determine the heavy metal contents of the plants, subsamples of the dried plants were ashed in a muffle furnace at 550 ^o^C for 6 h, and after cooled, the ashed samples were extracted with 1:1 hydrochloric acid solution and filtrated. The filtrates were measured for the concentrations of Zn, Pb, Cd, and Ni using an atomic absorption spectrophotometer (Z-2300, HITACHI).

### Chemical analysis and data analysis

For total heavy metal concentration determination, the samples of SS, SSB, stabilizers and soil were digested with HF-HNO_3_-HClO_4_, and concentrations of Zn, Pb, Cd, and Ni in the digested solution were determined using the AAS.

Available heavy metals in the samples were estimated by the DTPA-CaCl_2_-TEA extraction method [14]. Briefly, metals were extracted with a solution containing 0.005 M DTPA, 0.1 M triethanolamine (TEA) and 0.01 M CaCl_2_ at the soil: solution ratio of 1:5; pH of the resulting solution was adjusted to 7.30 with diluted HCl solution; concentrations of heavy metals in the extracts were determined using the AAS.

The chemical speciation (acid extractable fraction, reducible fraction, oxidizable fraction and residual fraction) for heavy metals from SS and SSBs were measure using the optimized BCR sequential extraction procedure [15].

Data were analyzed by ANOVA and differences between treatments were tested by Duncan's multi-range test (*P* = 0.05) using SAS software (version 8.2, SAS Institute, 2004).

## Results and discussion

### Total heavy metal concentration in SBBs

**Fig 2:**
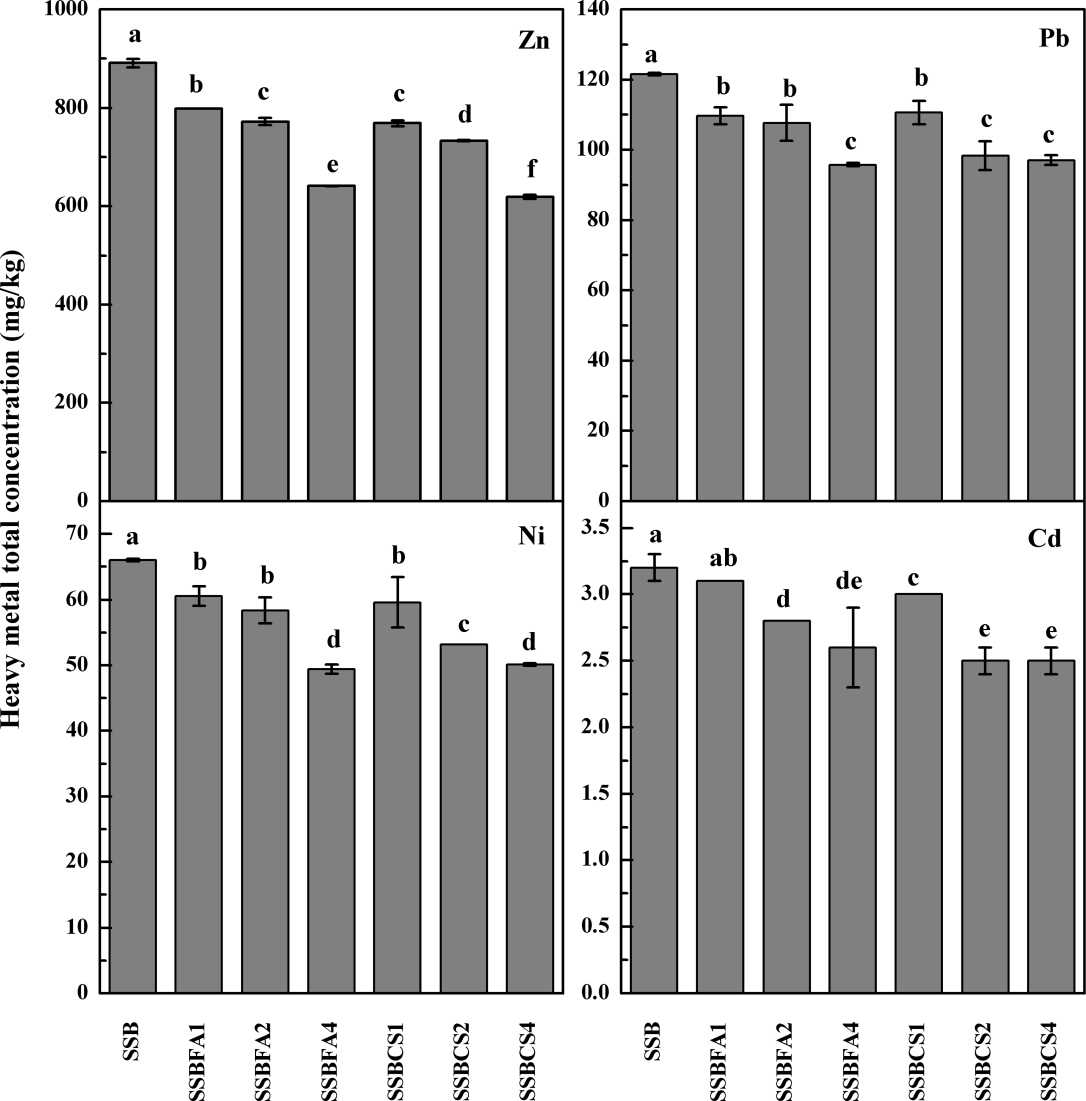
Total heavy metal concentrations in raw SSB (0% stabilizer) and SSB with FA or CS incorporated at 1%, 2% or 4%.

Carbonization raised total heavy metal concentrations in the SS due to loss of volatile components (**Table 1** and **Fig 2**). The contents of Zn and Ni increased by 16.6 and 29.5%, respectively; while those of Pb and Cd increased by 1.6% and 6.3%, respectively after SS carbonization. None of the heavy metal concentrations of the SSB exceeded the critical levels for sludge application in agriculture in China (GB4284-84). Similar results were reported by Koppolu et al. [16] that concentrations of Cu, Zn, Ni, Cr, and Co increased by four to six times in biochar relative to its feedstock.

Incorporation of stabilizers significantly decreased total heavy metal concentrations in the SSB (*P*<0.05), mainly due to dilution effect. However, the decrease varied between the two stabilizers, with CS being more effective than FA, likely because of partial loss of the FA stabilizer during the carbonization process.

### Available concentration and speciation of heavy metal in SBBs

**Fig 3:**
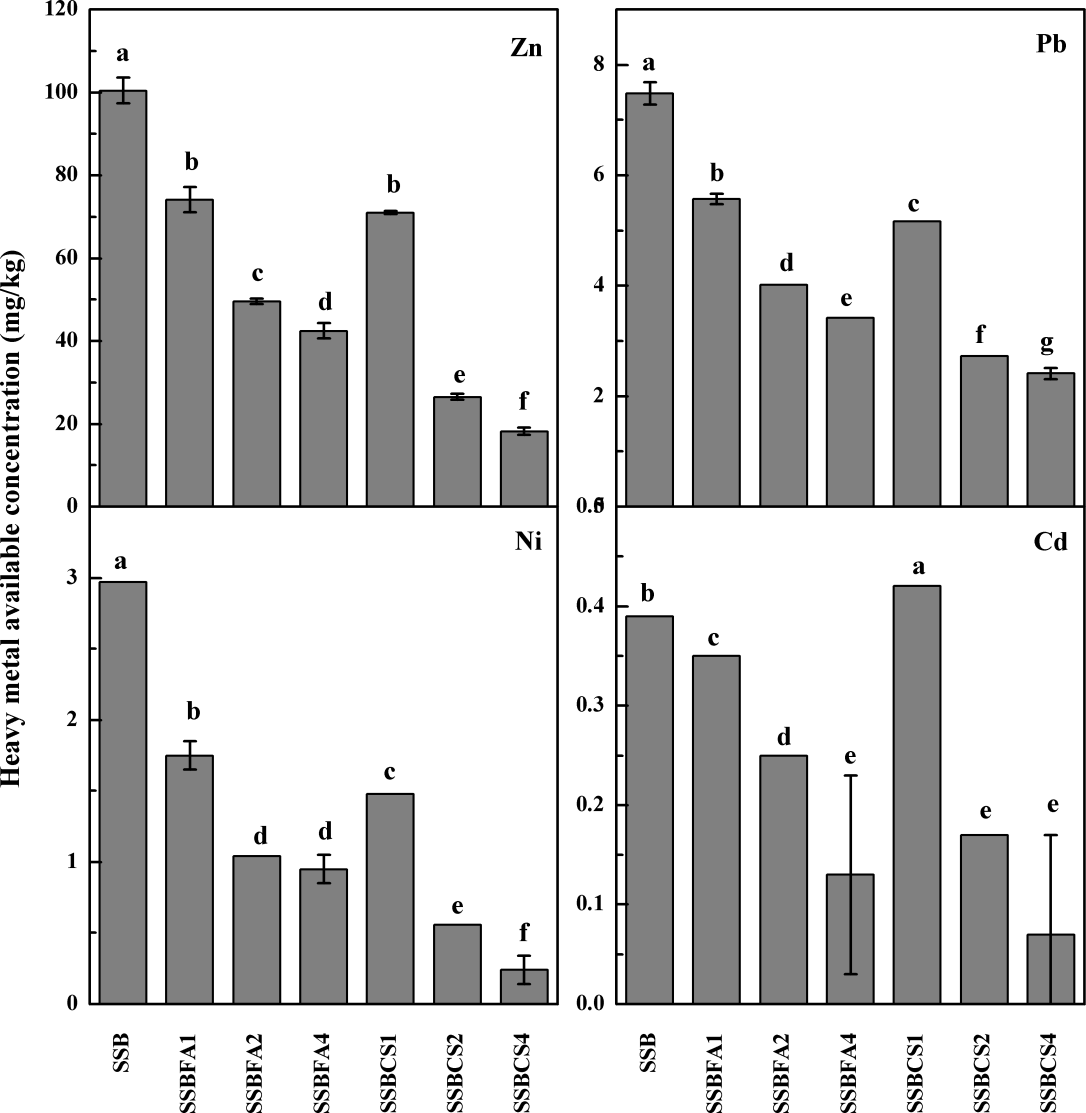
Available heavy metal concentrations (DTPA-CaCl_2_-TEA-available) in raw SSB (0% stabilizer) and SSB with FA or CS incorporated at 1%, 2% or 4%.

The environmental risk of a heavy metal is more related to its bioavailability or available concentration [17]. The major goal of stabilizer addition is to reduce availability of heavy metals in the SSB. As can be seen from **Fig 3**, available concentrations of Zn, Pb, Cd and Ni in the SSB, as estimated by the DTPA-CaCl_2_-TEA extraction method, were significantly decreased by stabilizer incorporation (*P*<0.05). The only exception was when CS was incorporated at 1%, the SSB had a higher available Cd concentration, as compared to control (SSB without stabilizer). Similar to total concentration, CS was more effective in decreasing available heavy metals than FA. The decreasing effect increased with increasing incorporation rate for both stabilizers (**Fig 3**). At 4% application rate, FA and CS decreased available heavy metal in SSB by 54–68% and 68–92%, respectively. To further investigate the stable states of heavy metals, the chemical speciation for heavy metals from SS and SSBs were analyzed using the optimized BCR sequential extraction procedure [15]. Sequential extraction methods may provide useful information on the potential mobility and association of heavy metals with different SBBs. It is confirmed that heavy metals in sulfuric and oxidizable state and residual state are stable states with lower envrionmental risk [18]. The relative distribution of heavy metals estimated by BCR extraction procedure in the SSB represented as percent of total concentrations are shown in **S2 Fig**. Compared with SS, the residual fraction for heavy metals in SSBs were significantly increased. With adding FA or CS, the sulfuric and oxidizable state and residual fraction for heavy metals in SSBs were increased at different extent, indicating the adding of FA or CS can promote forming stable state.

Therefore, incorporation of stabilizers prior to carbonization is a promising way to reduce availability of heavy metals in the SSB. In this study, available concentrations of Zn, Pb, Cd, and Ni were significantly lowered (*P*<0.05) with FA or CS incorporation. The reduction effect was more striking when the stabilizers were incorporated at a higher rate (**Fig 3**). The more striking reduction in available concentrations than in total concentration indicated that other mechanisms played a major role in lowering heavy metal bioavailability, in addition to dilution effect.

### Heavy metals uptake by plant

**Fig 4:**
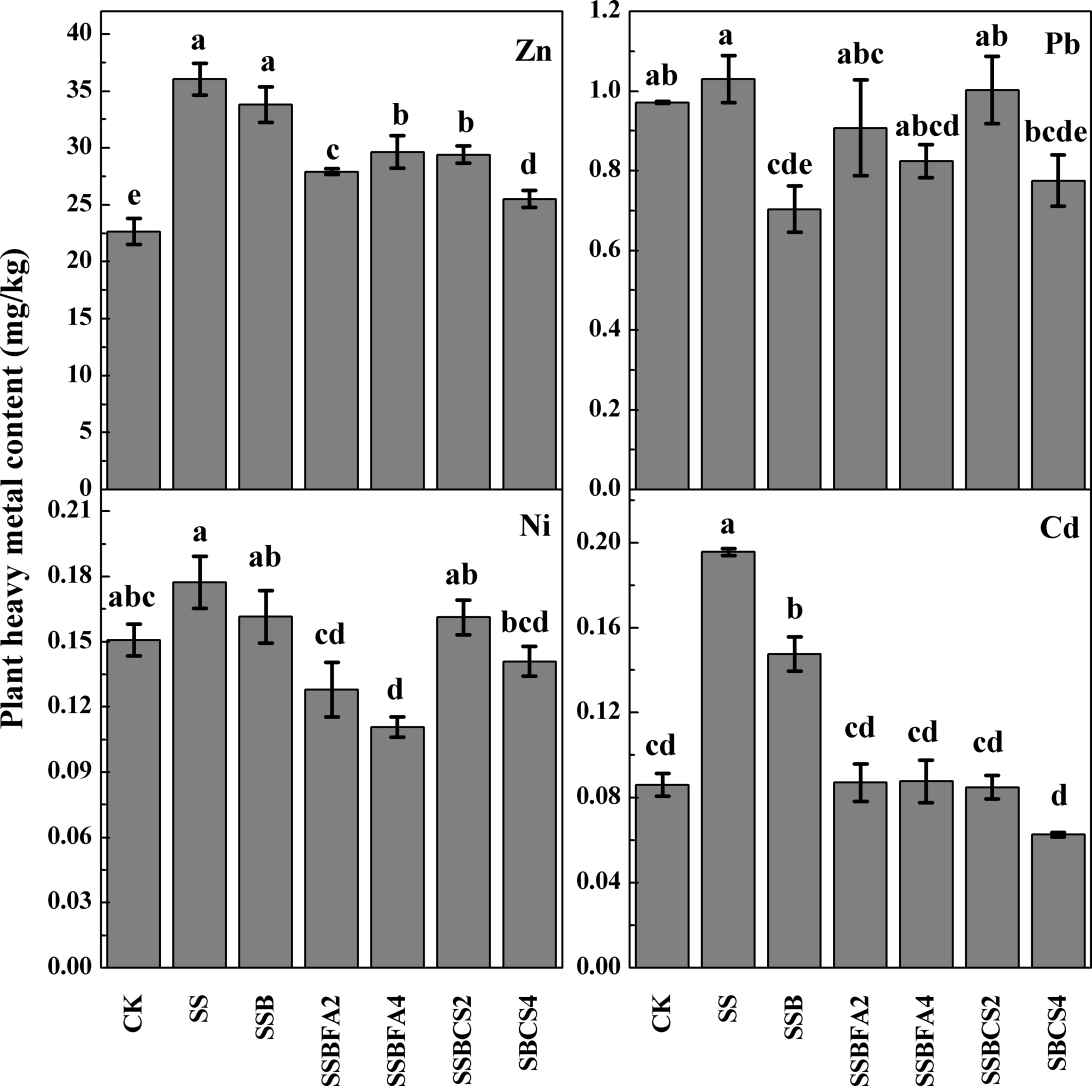
Heavy metal concentrations in plant received in different pot treatments with adding SS, SSB (0% stabilizer) and SSB with FA or CS incorporated at 2% or 4% (CK is no SS or SSB applied).

Amendment of SS alone generally increased concentrations of Zn, Pb, Ni, and Cd in plant, as compared to CK where only fertilizers were applied, indicating that heavy metals in the SS were available to plants (**Fig 4**). When SS was applied after carbonization as SSB (treatment SSB), less heavy metals were taken up by the plants, implying that carbonization reduced bioavailability of heavy metals in the SS. Based on the values of heavy metal available concentration in SSBFA1 and SSBCS1, the inhibition effects are still unsatisfied duo to the low dosage of stabilizer. Therefore, we decide not to study SSBFA1 and SSBCS1 treatments in the pot experiments. When the stabilizer incorporated SSB (SSBFA2, SSBFA4, SSBCS2, and SSBCS4) were applied, even less heavy metals were absorbed by the plants, indicated that incorporation of stabilizers further decreased bioavailability of heavy metals (Zn, Cd, Pb, and Ni) in SS and thus reduced environmental risk of SS for agricultural application.

**Fig 5:**
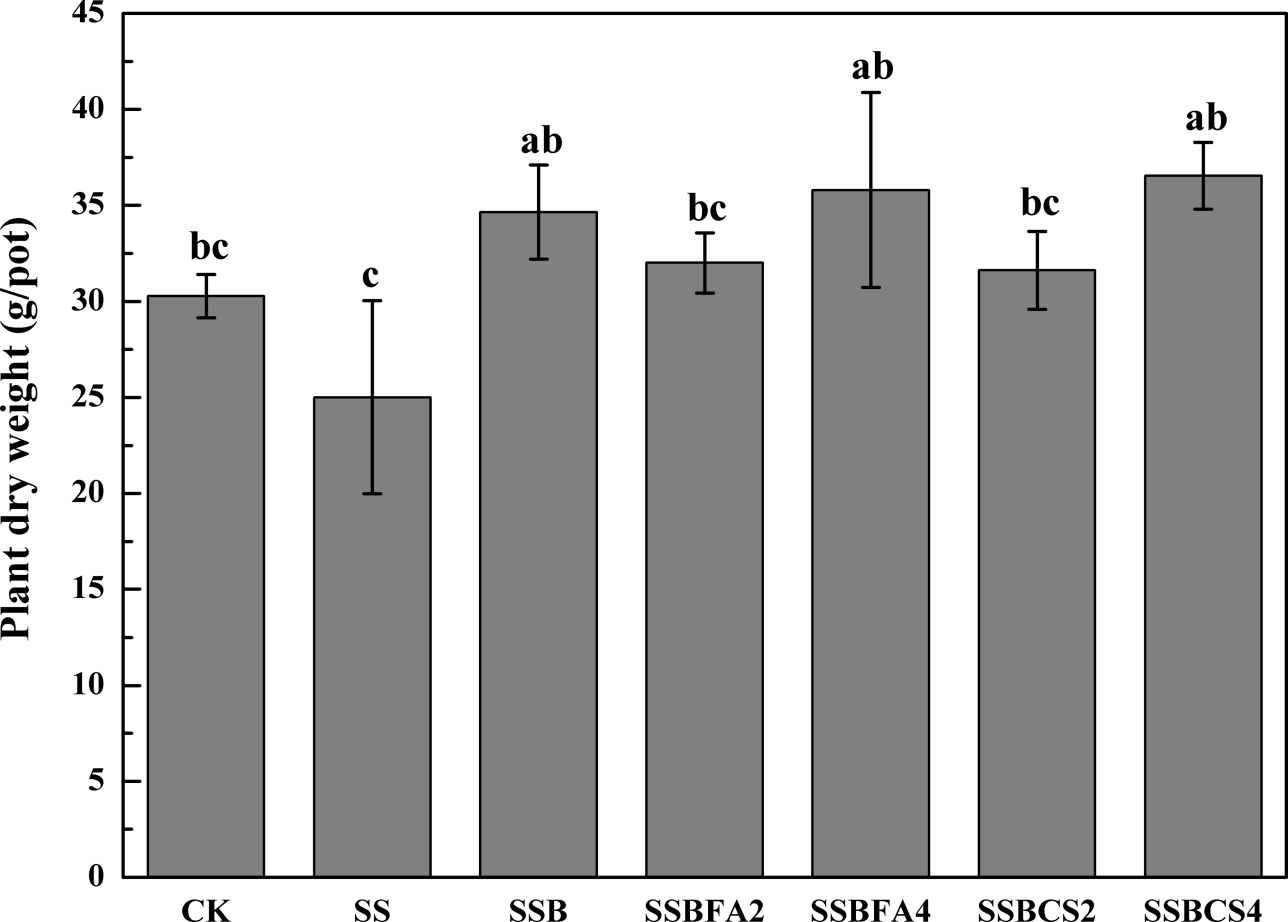
Plants biomass yields in different pot treatments with adding SS, SSB (0% stabilizer) and SSB with FA or CS incorporated at 2% or 4% (CK is no SS or SSB applied).

In treatment SS, plant dry weight was lower, as compared to CK (**Fig 5**), which may be attributed to negative influence of heavy metals in the SS applied. However, higher plant dry weights were achieved in the other five treatments with SSB applied than that in CK. This demonstrated that the stress of heavy metals from SS was mitigated by carbonization and incorporation of stabilizers [5,19]. In addition, higher plant dry biomass was obtained when FA or CS was incorporated at 4%, as compared to 2%, further demonstrating that effective mitigation of heavy metal toxicity could be accomplished by FA or CS incorporation at higher rates. SSBs can be used as a valuable soil amendment as it increases the organic matter content, the N, P and K content and can hinder the leaching of heavy metals present in raw sewage sludge [2, 3]. Therefore, SSB is a nutrient-rich material can be explored as a soil amendment and as a peat substitute for growing media formulation in biological agriculture and horticulture.

Although corn plants in this pot experiment were not grown to the harvest of corn cobs, which are the consumable part of corn, but many studies have demonstrate that there is a positive relationship between heavy metal concentration in crop straw and in grains, and between crop dry weight and grain heavy metal concentration [19]. That is, lower heavy metal contents can be expected in the corn grains harvested from the treatments of SBFA2, SBFA4, SBCS2, and SBCS4.

### Inhibition mechanism analyzed by FTIR and XPS

**Fig 6:**
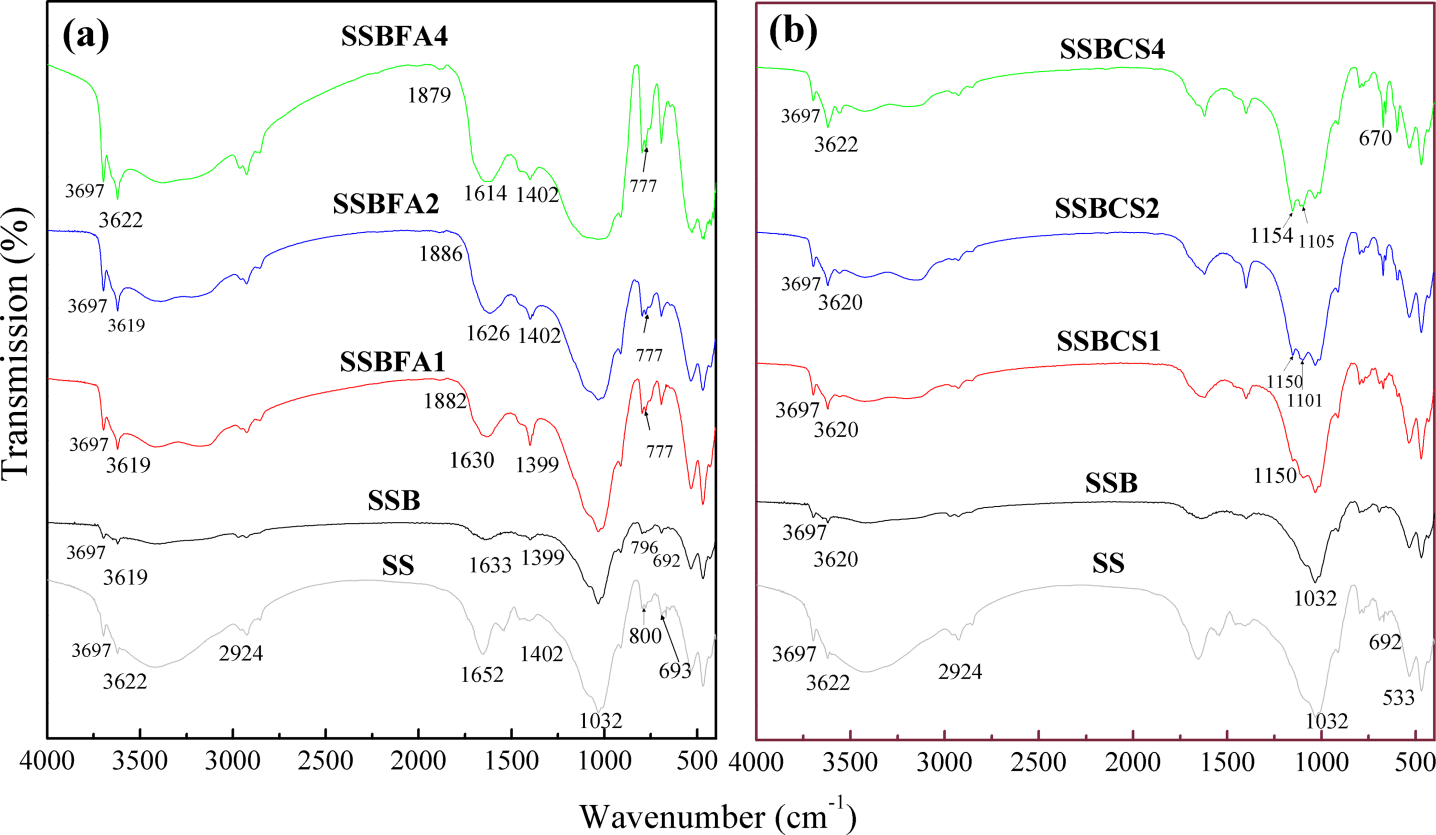
FTIR spectra of raw SS, SSB (0% stabilizer) and SSBs with FA (a) or CS (b) incorporated at 1%, 2% or 4%.

FTIR spectroscopy is a useful technique for the detection of functional groups in SS and SSBs. As can be seen in **Fig 6**, the peaks present in the FTIR spectra of SS were also observable in the FTIR spectra of SSB (0% stabilizer), but the intensity of the SSB peaks at 3600–3700 cm^-1^ and 1600–1700 cm^-1^ decreased dramatically after pyrolyzation at 350°C, owning to the dehydration reaction and dehydroxyl reaction. Compared to SSB, the spectra of SSBFA1, SSBFA2, and SSBFA4 showed prominent peaks between 3500–3700 cm^-1^, 2800–3000 cm^-1^, 1300–1500 cm^-1^, and 600–900 cm^-1^ (**Fig 6A**). These peaks are attributed to the stretching of OH, aliphatic or alicyclic C-H stretching, C = C and anti-symmetric COO^-^ stretching, and aromatic compounds, respectively [20]. This result indicated that the incorporation of FA into the SS prior to carbonization introduced additional functional groups of carboxyl, phenol, hydroxyl, amine and quinine groups. A new band appeared near the position at 1880 cm^-1^, which is assigned to the bending vibration of = C-H. Besides, with the increase of the amount of FA adding, the peak intensity at 1633 cm^-1^ that arises from the conjugated double bond C = C-C = C become stronger. These phenomena indicated that the adding of FA increase the aromatic hydrocarbons and unsaturated groups, which could enhance the chelate ability with heavy metals.

Besides the same prominent peaks between 3500–3700 cm^-1^, 2800–3000 cm^-1^, 1300–1500 cm^-1^, and 600–900 cm^-1^ as those in the spectra of SSBFAs, the spectra of SSBCSs showed other striking peaks between 1000–1150 cm^-1^ and 400–600 cm^-1^ (**Fig 6B**). The peaks between 1000–1150 cm^-1^ are assigned to the Si-O-Si asymmetric stretching mode [21,22] and S = O stretching vibration, and those between 400–600 cm^-1^ are attributed to inorganic matter, such as carbonate and silicate [23]. Carbonates and silicates are impurities of phosphogypsum. The difference FTIR spectra of SSB incorporated with stabilizers and SSB (spectral subtractions SSBFA4-SSB and SSBCS4-SSB) were compared with that of the respective stabilizers (FA and CS) are shown in **S3 Fig**. It is shown that the difference spectra of SSBCS4 and SSB displayed the typical peaks of CS at 3614 cm^-1^, 3546 cm^-1^, 1619 cm^-1^, 1152 cm^-1^, demonstrating the existence of CS in the SSBCS4 sample. On the contrary, the difference spectra of SSBFA4 and SSB differed dramatically with that of FA, which could be explained by the thermal instability of FA.

**Fig 7:**
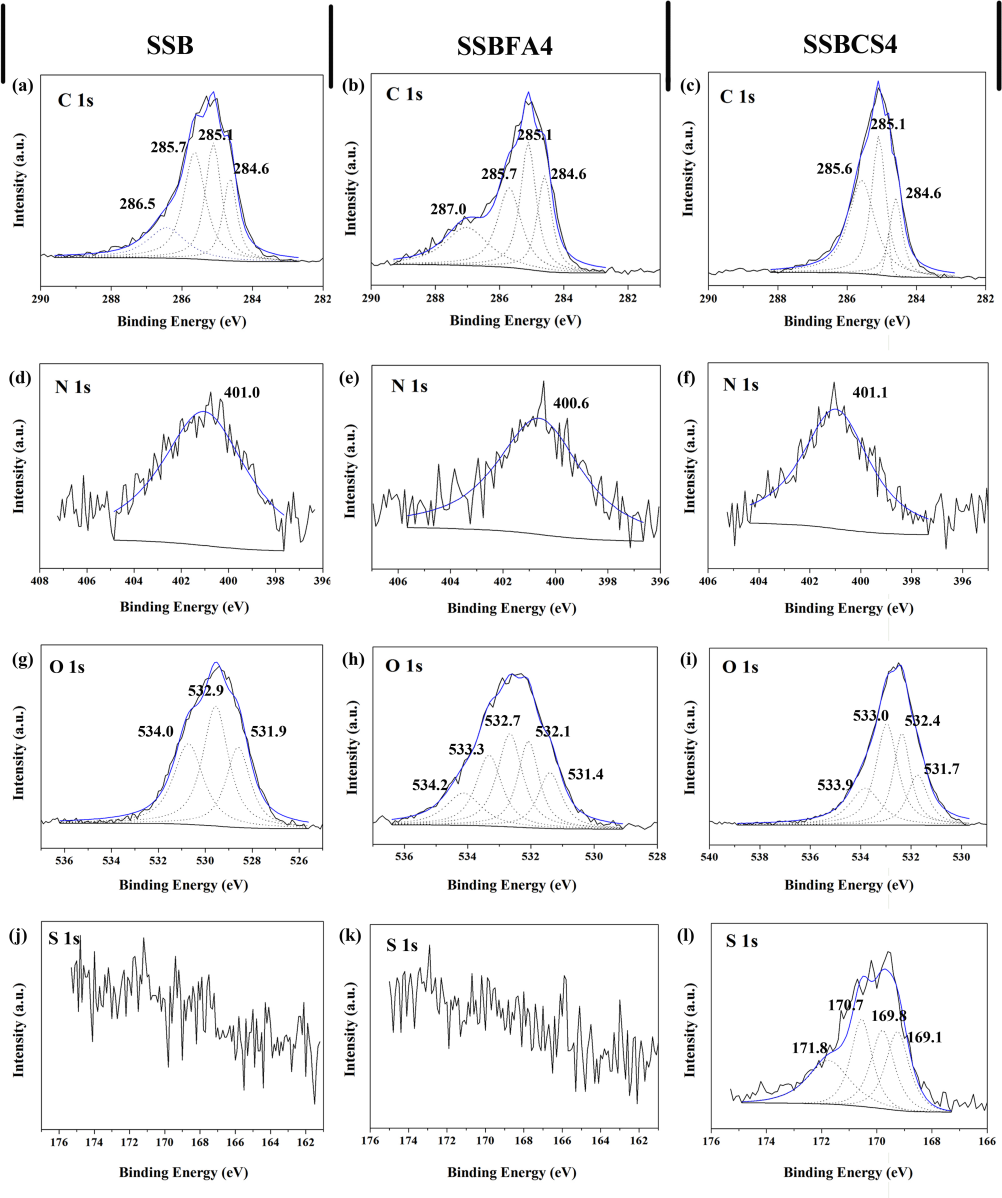
HR-XPS patterns of the samples: raw SSB (a), (d), (g), (j); SSBFA4 (b), (e), (h), (k); SSBCS4 (c), (f), (j), (l). (SSBFA4 and SSBCS4 are SSB incorporated with 4% FA and 4% CS, respectively).

To further decipher the inhibition mechanism of the heavy metals in SSBs, the HR-XPS of SSB samples were investigated. The details of C 1s, N 1s, O 1s and S 1s record for the samples are shown in **Fig 7**.

The C 1s XPS lines of the three samples were separated into four binding energy peaks. The positions of C 1s for the raw SSB sample (**Fig 7A**) are recorded at 284.6 eV (for–C-C or–CH), 285.1 eV (for–CH), 285.7 eV (for–CH or–C-N) and 286.5 eV (for–C-OH or–C-O), respectively. The C 1s peak shifted from 285.5 eV in the raw SSB to 287.0 eV in SBBFA4 sample (**Fig 7B**). The peak at 287.0 eV, which is assigned to the–C = O or–C = N groups, suggested some multifunctional organic ligands were formed in the process of the pyrolysis of sewage sludge with the addition of FA. These multifunctional organic ligands are beneficial to form heavy metal complexes, which will reduce the bioavailability of the heavy metals in SSB. The C 1s peaks in SSBCS4 sample (**Fig 7C**) got into a simplification with addition plenty of inorganic phosphogypsum. The N 1s peaks in the raw SSB (**Fig 7D**) and SSBCS4 sample (**Fig 7E**) showed a similar position (401 eV, for -C-N), suggesting that the N 1s chemical state were not changed in the process of addition phosphogypsum. However, the N 1s peaks in SSBFA4 sample shifted to 400.6 eV, which is assigned to–C-N or–C-N-Metal bonds, strongly confirming that more heavy metal complexes were formed [24]. The O 1s XPS lines of the raw SBB sample (**Fig 7G**) were separated into three binding energy peaks. The positions of O 1s for the raw SSB sample are recorded at 531.9.6 eV (for–C-O or–C-OH or Metal-OH), 532.9.1 eV (for–C = O) and 534.0 eV (for–C-O or–Metal-O), respectively. The peaks of O 1s got complicated after the FA treatment, and five peaks were observed in **Fig 7H**. The peaks presented some groups: Metal-OH at 531.4 eV, -C-O at 532.1 eV, -C = O at 532.7 eV, O = C-O at 533.3 eV, Metal-O or -C-O at 534.2. Some of the oxygen-containing groups constituted multifunctional organic ligands, which are beneficial to form heavy metal complexes. For SBBCS4 sample, some inorganic and organic oxygen-containing groups were observed: 531.7 eV for M-OH or N-C-O, 532.4 eV for SO_4_^2-^, 533.0 eV for P-O-P or -C = O, and 533.9 eV for PO_3_^-^ or P-O-P or C-O-C. The P element in SSBCS4 is mainly come from phosphogypsum.

Finally, as can be seen from **Fig 7J** and **Fig 7K**, no S 1s signal was observe in XPS lines for the raw SSB sample and SSBFA4 sample due to the low content of S element. For the sample of SSBCS4 (**Fig 7L**), some important chemical states of S were found: 169.1 eV for SO_4_^2-^, 169.8 eV for S^2-^ or R-SO2-O-R, 170.7 eV for C-O-S, and 171.8 eV for FeS. This finding indicate convincingly that the adding of phosphogypsum into sewage sludge could reduce the bioavailability of heavy metals by forming metal sulfide after pyrolysis treatment.

In general, it is well known that FA has plenty of functional groups, including carboxyl, phenol, hydroxyl, amine and quinine groups, and its incorporation increased such functional groups in the SSBs as evident from the FTIR spectra (**Fig 6**). These groups may have chelated with heavy metals [25], thus reducing bio-availabilities of the heavy metals in the SSB. The incorporation of CS substantially increased Ca^2+^ and SO_4_^2-^ concentrations in SSB; SO_4_^2-^ can replace HPO_4_^2-^/H_2_PO_4_^-^ from surface of SSB. Both sulfate and phosphate can react with Pb, Cd, Zn, and Ni to form water insoluble compounds such as PbSO_4_ (anglesite), Pb_3_(PO_4_)_2_/Zn_3_(PO_4_)_2_/Cd_3_(PO_4_)_2_ during the carbonization. As a result, the availability of heavy metals in the SSB was lowered [17]. FA or CS addition may provide a new applicable method for enhancing the heavy metals immobilization in sludge by carbonization at 350°C, providing a safe and sustainable treatment method for heavy metals-contaminated sludge before land use.

## Conclusions

It has been controversial to use SS as soil amendment in agriculture even though it is rich in organic C and nutrients. Potential impact of heavy metals in SS on soil quality and food safety has been a public concern worldwide. Carbonization appears to be effective in increasing the percentage of heavy metals in sulfuric and oxidizable state and residual state, and reducing mobility and plant-availability of heavy metals in SSB. Incorporation of selected stabilizers such as FA and CS during the sludge carbonization can further enhance the heavy metals immobilization and lower plant-availability of heavy metals in the SSB. This finding is conductive to a safe and beneficial method for land application or horticulture as a peat substitute of sewage sludge. However, long-term experiments are still needed to monitor the fate of heavy metals in SSB.

## Supporting information

S1 Fig Loss on thermal conversion for SS samples. At 350 for 1 h, the weight loss is approximately 20%.
(DOCX)

S2 Fig Chemical speciation for heavy metals in raw SSB (0% stabilizer) and SSB with FA or CS incorporated at 1%, 2% or 4%. The sulfuric and oxidizable oxidizable fraction and residual fraction of the heavy metals in SSBs were further increased after incorporation with stabilizers.
(DOCX)

S3 Fig **The difference FTIR spectra of SSB incorporated with stabilizers and SSB (SSBFA4-SSB in a and SSBCS4-SSB in b) were compared with that of the respective stabilizers (FA and CS).** The changes of the functional groups in CS and FA after incorporation into SSBs are different.
(DOCX)
