# Supplementary material for: Inhibition of the bioavailability of heavy metals in sewage sludge biochar by adding two stabilizers
Source: PLoS One. 2017 Aug 23;12(8):e0183617. doi: 10.1371/journal.pone.0183617 (PMC5568343; doi:10.1371/journal.pone.0183617)
Supplement: S1 Fig — At 350 for 1 h, the weight loss is approximately 20%. (DOCX) [file pone.0183617.s001.docx]

**S1 Fig Loss on thermal conversion for SS samples.**

Considering that lowering the calcination temperature can reduce the energy and financial cost, we have chose a relatively low calcination temperature of 350 for 1 h. At such temperature, the weight loss is approximately 20%. Since the weight loss is relatively low, the heavy metals in the samples will not be significantly affected by the thermal conversion. The dosage of stabilizers added is small, hence the application of the stabilizers also has little effect on the weight loss of the samples.
