# Supplementary material for: Inhibition of the bioavailability of heavy metals in sewage sludge biochar by adding two stabilizers
Source: PLoS One. 2017 Aug 23;12(8):e0183617. doi: 10.1371/journal.pone.0183617 (PMC5568343; doi:10.1371/journal.pone.0183617)
Supplement: S2 Fig — The sulfuric and oxidizable oxidizable fraction and residual fraction of the heavy metals in SSBs were further increased after incorporation with stabilizers. (DOCX) [file pone.0183617.s002.docx]

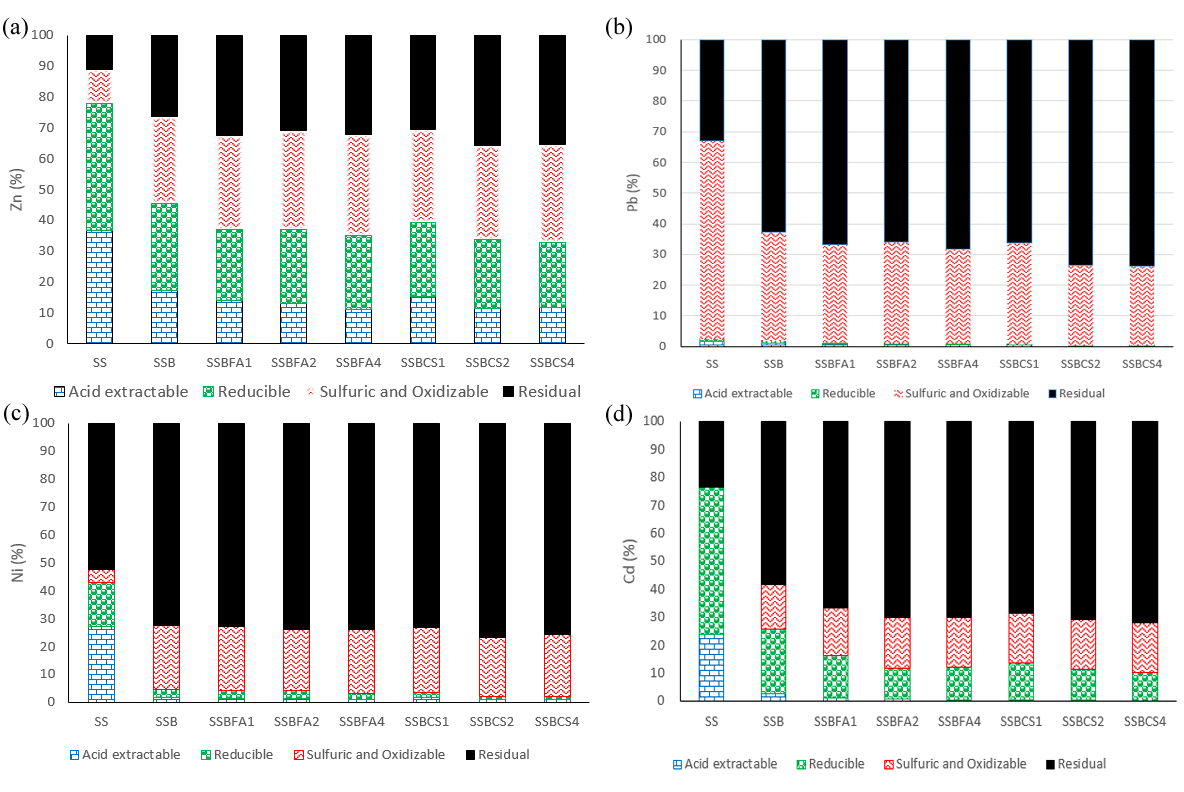


**S2 Fig Chemical speciation for heavy metals in raw SSB (0% stabilizer) and SSB with FA or CS incorporated at 1%, 2% or 4%.**

Sequential extraction methods may provide useful information on the potential mobility and association of heavy metals with different SBBs. The chemical speciation experiment can isolate heavy metals in different fractions including acid extractable fraction, reducible fraction, sulfuric and oxidizable fraction and Residual fraction. The results indicated that after pyrolyzation the percentage of heavy metals (except for Pb) in sulfuric and oxidizable state and residual state were significantly increased, hence the mobility of the heavy metals in SSBs was decreased. And the sulfuric and oxidizable oxidizable fraction and residual fraction of the heavy metals in SSBs were further increased after incorporation with stabilizers. Oxidizable oxidizable state and residual state were confirmed as stable states for heavy metals.
