# Supplementary material for: Inhibition of the bioavailability of heavy metals in sewage sludge biochar by adding two stabilizers
Source: PLoS One. 2017 Aug 23;12(8):e0183617. doi: 10.1371/journal.pone.0183617 (PMC5568343; doi:10.1371/journal.pone.0183617)
Supplement: S3 Fig — The difference FTIR spectra of SSB incorporated with stabilizers and SSB (SSBFA4-SSB in a and SSBCS4-SSB in b) were compared with that of the respective stabilizers (FA and CS). The changes of the functional groups in CS and FA after incorporation into SSBs are different. (DOCX) [file pone.0183617.s003.docx]

**S3 Fig The difference FTIR spectra of SSB incorporated with stabilizers and SSB (SSBFA4-SSB in a and SSBCS4-SSB in b) were compared with that of the respective stabilizers (FA and CS)**

It was shown that the difference spectra of SSBCS4 and SSB displayed the typical peaks of CS at 3614 cm^-1^, 3546 cm^-1^, 1619 cm^-1^, 1152 cm^-1^, demonstrating the existence of CS in the SSBCS4 sample. On the contrary, the difference spectra of SSBFA4 and SSB differed dramatically with that of FA, which could be explained by the thermal instability of FA. The spectral subtractions are interesting, and could provide some information on the general characteristics of the samples, however, the results are easily influenced by multiple factors and some peaks maybe no sense.
